# The Past, Present, and Future of Human Centromere Genomics

**DOI:** 10.3390/genes5010033

**Published:** 2014-01-23

**Authors:** Megan E. Aldrup-MacDonald, Beth A. Sullivan

**Affiliations:** 1 Department of Molecular Genetics and Microbiology, Duke University Medical Center, Durham, NC 27710, USA; E-Mail: megan.aldrup@duke.edu; 2 Division of Human Genetics, Duke University, Durham, NC 27710, USA

**Keywords:** alpha satellite, higher order repeat, CENP, heterochromatin, human artificial chromosome, dicentric, chromosome truncation, transcription, tet operon

## Abstract

The centromere is the chromosomal locus essential for chromosome inheritance and genome stability. Human centromeres are located at repetitive alpha satellite DNA arrays that compose approximately 5% of the genome. Contiguous alpha satellite DNA sequence is absent from the assembled reference genome, limiting current understanding of centromere organization and function. Here, we review the progress in centromere genomics spanning the discovery of the sequence to its molecular characterization and the work done during the Human Genome Project era to elucidate alpha satellite structure and sequence variation. We discuss exciting recent advances in alpha satellite sequence assembly that have provided important insight into the abundance and complex organization of this sequence on human chromosomes. In light of these new findings, we offer perspectives for future studies of human centromere assembly and function.

## 1. Introduction

The centromere is the chromosomal locus that controls chromosome segregation during cell division. Visually, the centromere appears on metaphase chromosomes, at least in metazoans that have excellent cytology, as a primary constriction. This is also the site of kinetochore assembly, the multi-protein structure that forms to coordinate attachment to and movement of chromosomes along microtubules. The proteins associated with centromeres are conserved among species, consistent with the functional significance of the locus. A surprising feature of centromeres is that the DNA sequences present at the locus are dissimilar, not only among organisms but often within the same organism. However, protein components of centromeres are shared among species, suggesting an epigenetic basis for centromere assembly. Such centromere proteins (CENPs) include CENP-A, CENP-C, and CENP-T that are important for structural and functional aspects of the centromere and kinetochore. CENP-A is of particular significance since it is a histone H3 variant that contributes to specialized chromatin at centromeres. The Holliday Junction Recognition Protein HJURP and its fungal homolog Scm3 are chaperones that direct the loading of CENP-A into chromatin primed by the Mis18 complex and ensure propagation of epigenetically marked centromeric nucleosomes (reviewed by [1,2]).

Despite the lack of sequence identity, many centromeres are located in regions of repetitive DNA or satellites. In humans, repetitive alpha satellite DNA defines the centromere. The sequence basis of centromere identity is widely debated, since variant centromeres have been identified in humans and other organisms. These unusual centromeres include neocentromeres, new centromeres that are formed on unique or non-centromeric DNA sequences [3,4]. Dicentric human chromosomes, those chromosomes that are formed by fusion or translocation, have two regions of centromeric DNA, but often only one is the site of kinetochore formation. In these instances, the alpha satellite DNA appears to be neither necessary nor sufficient for centromere function. Nevertheless, other evidence exists that supports the importance of DNA sequence in centromere formation in humans, particularly *de novo* centromere assembly. In this review, we will discuss advances in our understanding of human centromeric DNA, from the discovery of human centromeric sequences through integration of physical and genetic maps of centromeres during the Human Genome Project era to the first centromeric genome assemblies that are only now emerging.

## 2. Alpha Satellite DNA: Discovery, Organization, and Variation

**Figure 1:**
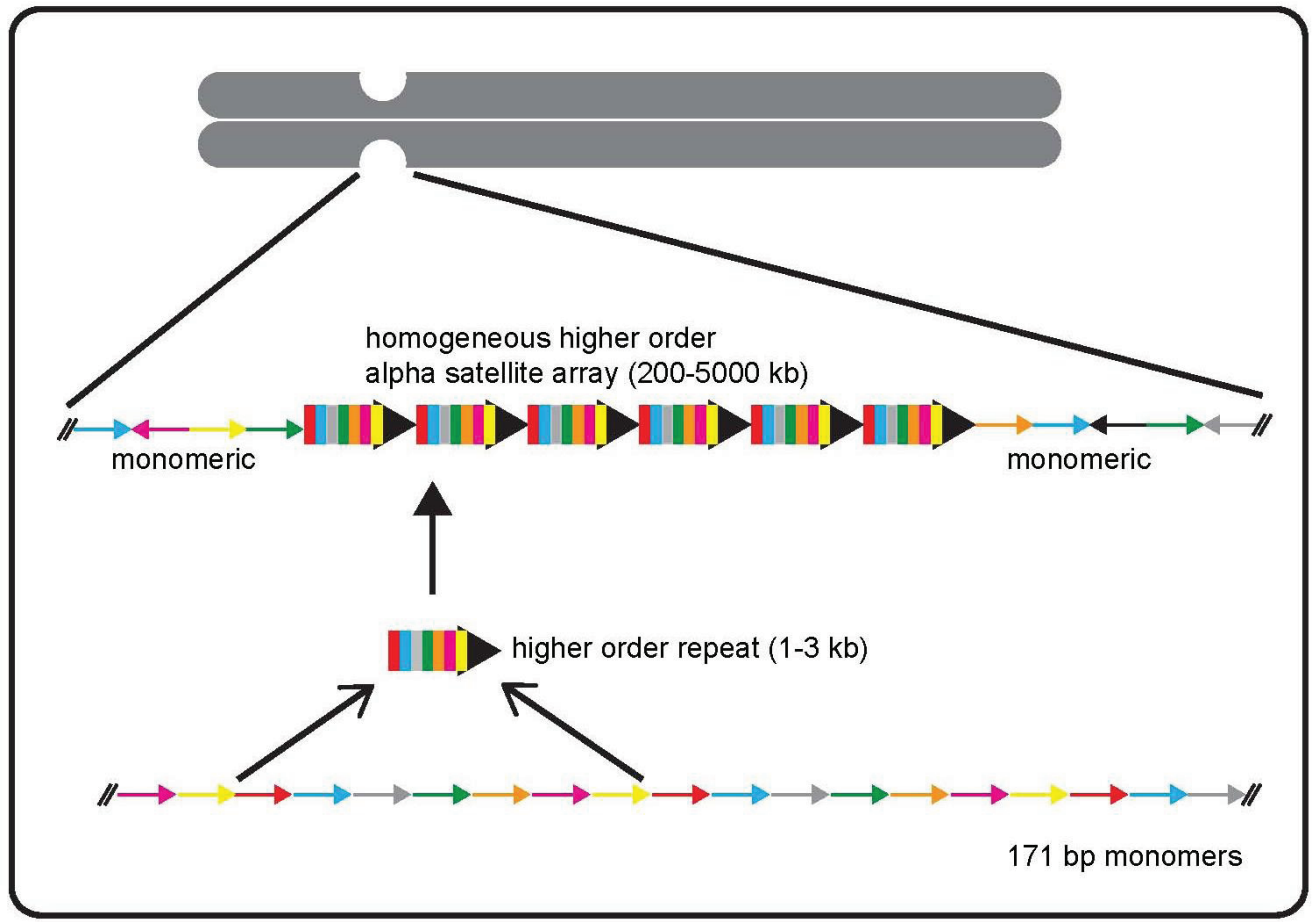
The genomic organization of human centromeres. The primary sequence at human centromeres is alpha satellite DNA that is based on 171 bp monomers (colored arrows) organized in a tandem head-to tail fashion. The monomeric sequences differ by as much as 40%. A set number of monomers give rise to a higher order repeat (colored bars with black arrowhead) and confer chromosome-specificity. Higher order repeats are themselves reiterated hundreds to thousands of times, so that the alpha satellite arrays are highly homogenous and span several hundred kilobases to several megabases. Unordered monomeric alpha satellite DNA flanks the higher order arrays, becoming progressively more divergent farther away from centromeric core.

Human centromeres, and in fact most primate centromeres, are composed of alpha satellite DNA [5]. This sequence is thought to be important for centromere function since it is present at the primary constriction of all human chromosomes. It comprises up to 5% of the genome. Alpha satellite is based on a 171 bp monomer arranged in a tandem, head-to-tail fashion. Individual monomers share 50%–70% sequence identity. An integral number of monomers give rise to a higher order repeat (HOR) unit that is itself repeated in a largely uninterrupted fashion so that within a given centromeric locus, the alpha satellite array can span from 250 to 5,000 kb. Such re-iteration of the HOR gives rise to a homogenized alpha satellite array in which the HORs differ in sequence by only a few percent (Figure 1), even though the constituent monomers show much less sequence similarity [6,7]. Some monomers within the HORs contain a 17 bp sequence called the CENP-B box, a motif that is recognized by the DNA-binding centromere protein CENP-B [8]. Outside of the higher order arrays, monomers are randomly arranged and span the region between the homogeneous array and the chromosome arm [9].

**Figure 2:**
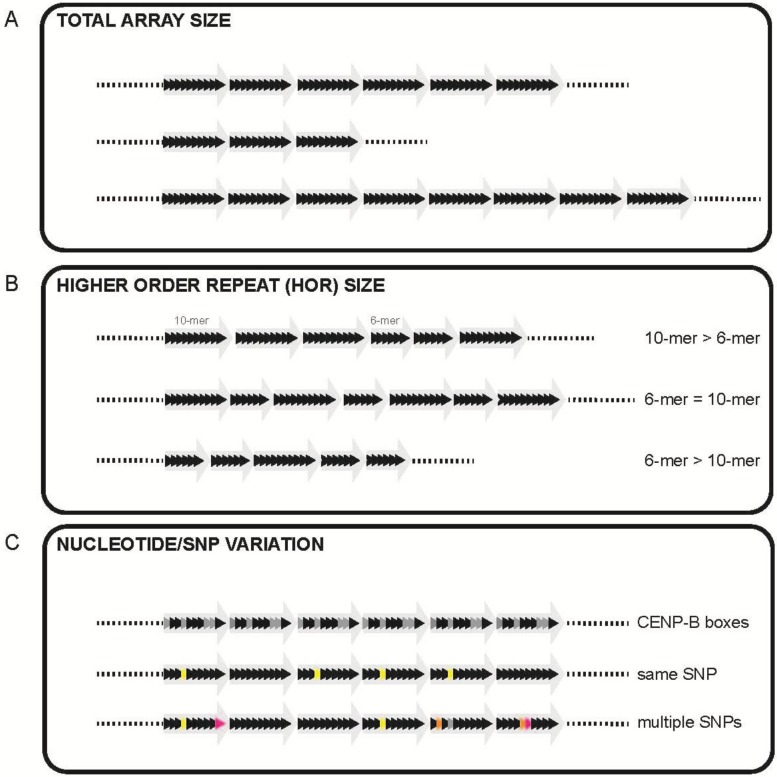
Heterogeneity of alpha satellite DNA. The alpha satellite DNA at centromeres exhibits several types of polymorphism. (**A**) Total array size, defined by the number of higher order repeats (HOR; gray arrows), varies between homologues and among individuals; (**B**) The same alpha satellite array from a given chromosome type can contain HORs of different sizes. In addition, the number of each HOR variant can vary. For example, an alpha satellite array can contain a mixture of 10-mers and 6-mers, with a greater number of 10-mers. Another array from the same chromosome in a different individual might have an equal number of 10-mers and 6-mers or, alternatively, more 6-mers than 10-mers; (**C**) Alpha satellite DNA can also vary at the level of monomer (black arrowheads) type and arrangement. Some monomers (gray arrowheads) contain a specific sequence element called the CENP-B box. Others monomers can contain identical nucleotide changes or SNPs (yellow arrowheads) within the same array. Multiple SNPs (hot pink, orange, gray, yellow arrowheads) can be present in the same HOR or distributed across an alpha satellite array. Each type of variation (array size, HOR size, SNPs) is not mutually exclusive and all contribute to the heterogeneity of alpha satellite DNA in the human population.

Variation within the alpha satellite is common and complex. Each chromosome type is defined by an alpha satellite array in which the multimers of the HOR contain a particular number of tandem monomers [7,10,11]. The homogeneity of HORs of the same monomer number makes the alpha satellite array chromosome-specific and distinguishable from related sequences at other centromeres. Certain chromosomes share greater homology of HORs based on monomer subtypes and organization, allowing them to be classified into one of three suprachromosomal families [12]. Diverged monomeric alpha satellite falls into two additional suprachromosomal families [13]. On a given chromosome type, the number of times the HOR is re-iterated is heterogeneous, spanning hundreds to thousands of copies. Consequently, total array size on a given chromosome varies between homologs and among individuals (Figure 2) [14,15,16,17,18]. Although array sizes can be as small as a few hundred kilobases or as large as five megabases [16,19,20], the range appears to be less extensive on a particular chromosome type [21]. Array size polymorphisms are largely stable through meiosis since they can be efficiently tracked through multigenerational families [17]. These polymorphisms make alpha satellite a useful centromeric marker for tracking inheritance of individual chromosomes.

Additional alpha satellite variation exists at the level of the HOR. On a given chromosome, the primary HOR unit can exhibit size polymorphisms that are most likely the result of deletions caused by unequal exchange [22,23]. Human chromosome 17 is a good example of HOR polymorphism within the D17Z1 alpha satellite array. The predominant HOR on D17Z1 is a 16-monomer (16-mer) [18,22]. However, less prevalent 15-mer and 14-mer HORs are present on many D17Z1 arrays, as well as 13-mers and 12-mers [22,24]. Within this group, the 13-mer is the most abundant after the 16-mer. These size polymorphisms create centromeric haplotypes, with the 16-/15-/14-mer comprising a haplotype found on 65% of chromosome 17 s and the additional 13-mer present on 35% of chromosome 17 s [22,25]. A recent study that evaluated centromere assembly on multiple chromosome 17 s suggested that HOR variants might have different functional capacities [26]. This possibility, however, remains to be formally tested in an independent functional assay.

## 3. Functional Studies that Have Defined Genomic Centromeres

**Figure 3:**
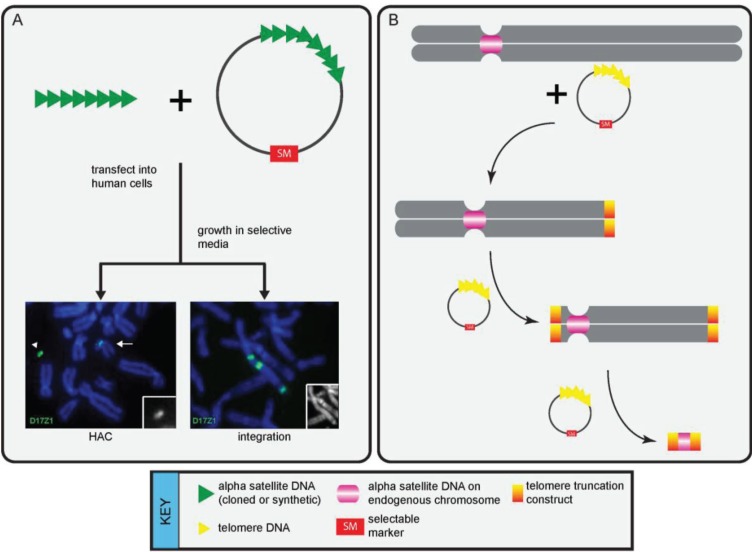
Minichromosome-based assays defining alpha satellite as the functional human centromere. (**A**) In the late 1990s, human artificial chromosome (HAC) assays (bottom up approach) were developed to test the ability of alpha satellite DNA to form *de novo* centromeres. Synthetic or clone arrays of alpha satellite DNA, such as D17Z1 from human chromosome 17 (green), were cloned into bacterial or plasmid (P1) artificial chromosome (BAC/PAC) vectors containing selectable marker genes (SM). The chromosome assembly constructs were introduced by transfection into human cells. In approximately half of the cells, an autonomous *de novo* chromosome (arrowhead) was produced, consisting of the same alpha satellite DNA (D17Z1, green, as shown) as the parental chromosome (arrow). Inset shows DAPI (DNA) staining of HAC. In the other proportion of transfected clones, the alpha satellite assembly BAC/PAC vector does not make a HAC, but integrates once or multiple times (as shown) into one or more chromosomes. In these instances, the alpha satellite DNA does not recruit any, or all, centromere proteins and is not a functional centromere. Inset shows DAPI (DNA) stained chromosome that contains multiple insertions of D17Z1. (**B**) In a complementary top-down approach, existing chromosomes (X and Y) were systematically deleted using plasmid constructs containing mammalian telomeric sequence (yellow arrowheads). These experiments yielded partially deleted chromosomes with integrated telomeres (red-orange-yellow rectangles) that were progressively deleted. Mitotic chromosome segregation of these minichromosomes was used as a measure of chromosome stability. Based on the molecular composition of the stable minichromosomes that were recovered, alpha satellite DNA (pink oval) was defined as the minimal sequence required for centromere function.

The strongest evidence for implicating alpha satellite DNA in human centromere function came from two lines of chromosome engineering experiments that took “bottom up” and “top down” approaches (Figure 3). In the “top down” strategy, telomere-mediated chromosomal truncation was used to modify the X chromosome or Y chromosome, some of which had been transferred to either rodent somatic cell hybrids or DT40 chicken cells (Figure 3B) [27,28]. Because DT40 cells are proficient in homologous recombination, targeted seeding of the telomere truncation constructs accelerated the deletion process. Multiple rounds of telomere truncation generated a series of deleted chromosomes, each containing less X or Y chromosome material (Figure 3B). The stability of the minichromosomes was monitored and those that maintained the least amount of the original chromosome but were still mitotically stable were concluded to contain the minimal sequence(s) necessary for centromere function. In both truncated X and Y chromosomes, minichromosomes containing alpha satellite DNA arrays DXZ1 and DYZ3, respectively, equated with the most stable linear minichromosomes. These studies strongly implicated alpha satellite DNA as the sequence that corresponds to centromere function and chromosome stability.

However, it could be argued that in the top-down studies the centromere, once established on any sequence, stays at that sequence, and does not shift with truncation. At the same time, pioneering experiments were being developed by two groups to take a “bottom up” approach to define the sequences required for centromere function. In these studies, alpha satellite sequences were introduced into linear yeast artificial chromosome (YAC) or circular bacterial artificial chromosome (BAC) vectors (Figure 3A). Hunt Willard’s group created first generation artificial chromosomes from synthetic alpha satellite arrays [29]. One higher order repeat from D17Z1 (chromosome 17) or DYZ3 (chromosome Y) was amplified through successive rounds of directional cloning to yield a 1Mb array that was inserted into a BAC vector. Introduction of these artificial chromosome assembly constructs by liposome-mediated transfection into the HT1080 cell line yielded clones that contained a microchromosome or human artificial chromosome (HAC). The HACs recruited centromere proteins and were stable in mitosis for at least 6 months. Careful analysis of the HACs showed that the D17Z1 HACs were completely derived from the input construct. However, the DYZ3-derived HAC had acquired additional alpha satellite sequences from host chromosomes. The functional significance of the inability of DYZ3 to form a functional HAC containing only Y centromere sequences was not fully appreciated at the time. Subsequent studies shed light on the correlation between DYZ3 sequence and its competence for *de novo* centromere assembly (see below).

At the same time that the Willard group was creating HACs from synthetic alpha satellite DNA, Howard Cooke’s and Hiroshi Masumoto’s groups were collaborating to clone large alpha satellite arrays from human chromosome 21 into linear YAC vectors. In their studies, the higher order array α21-I and the unordered monomeric array α21-II were introduced into HT1080 cells and compared for *de novo* centromere competency [30]. Only YACs containing the α21-I HOR array were capable of forming mitotically stable HACs that properly assembled centromere proteins. These innovative studies complemented those of the Willard group, and contributed important structure-function information that implicated HOR alpha satellite as a preferred substrate for *de novo* centromere assembly. In the time that has elapsed since these groundbreaking experiments, additional studies have established HACs as models for testing the genomic (and epigenetic) requirements for *de novo* centromere assembly and function. Circular BAC and PAC vectors, rather than linear YACs, are the most useful assembly vectors and are associated with high rates of HAC formation [31,32]. Not all alpha satellite arrays translate to HAC formation. Y chromosome alpha satellite DNA (DYZ3) lacks CENP-B boxes and is unable to efficiently form *de novo* centromeres on HACs [29,32]. Furthermore, arrays containing mutated CENP-B boxes cannot form *de novo* HACs [33]. Thus, the presence of CENP-B binding sites is required for centromere assembly. This has been a perplexing finding, given that the Y chromosome clearly assembles a functional centromere and recruits essential centromere proteins. These findings hint at key differences between *de novo* versus established centromere function that are not well understood.

Initial studies that tested the ability of alpha satellite to nucleate functional centromeres introduced cosmids containing human alpha satellite DNA from chromosome 17 into African green monkey (AGM) cells [34]. These experiments did not result in supernumerary chromosomes or HACs, but instead, integration of the alpha satellite construct into AGM chromosomes (Figure 3A). Indeed, up to 60% of HAC constructs introduced into human cells integrate into the genome rather than forming an independent chromosome. While some might point out that this argues against the case for sequence-dependent centromere assembly, another interpretation is that *de novo* chromosome assembly and *de novo* centromere formation are two different processes. Indeed, some integrated alpha satellite arrays recruit centromere proteins [34,35], although they may not retain some or all of the proteins long-term. At the very least, both integrated and free-lying HAC studies suggest that alpha satellite provides sequence information for some aspects of centromere function.

Contemporary studies are now using centromere-based chromosome engineering to create a new generation of HACs that contain alpha satellite in addition to tetracycline operator (tetO) sequences [36]. The tetO sequences are bound with high affinity by the tet repressor (tetR) that can be fused to different proteins in order to manipulate the chromatin or protein composition of the HAC [37,38]. In this way, centromere assembly on the alpha satellite can be enhanced or inhibited, the long-term stability of the HAC can be monitored by tethering tetR fluorescent protein fusions, and expression of genes included on the HAC can be tested [39].

## 4. Centromere Regions in the Human Genome Project Era

As the understanding of the relationship between alpha satellite DNA and centromere function emerged at the end of the 20th century, it led to a call for the identification and mapping of functional centromere sequences [40]. However, the nature of alpha satellite, with its megabase-scale regions of higher-order repetitive structure, made it highly refractory to sequencing and assembly [41]. As the Human Genome Project (HGP) rapidly increased the sequence information available for testing human genome function, gains were largely not seen at the pericentromeres and centromeres of most human chromosomes. A 1998 plan for the project that outlined the HGP’s goal for a 2001 working draft and a 2003 final draft acknowledged that “the small proportion of highly repeated sequence represented by the centromeres and other constitutive heterochromatic regions of the genome” might not be included in the final reference assembly [42]. A contemporary perspective on the plan warned of the possibility that potentially important duplications and tandem repeats would be “swept under the carpet”. There was a repeated call for at least some centromeric regions to be characterized in order to confirm that their structure was as homogenous as originally claimed [43]. But again, due to the computational complexity required to accurately assemble such highly repetitive regions, few labs attempted to close these sequence gaps [44,45,46]. A decade later, multi-megabase-sized gaps remain at the centromeres of most chromosome assemblies. This problem is not exclusive to the human genome, since centromere and pericentromere sequence gaps in other organisms such as mouse and Drosophila remain unclosed [47,48,49,50]. Only in the past year have advances in sequencing technologies and innovative computational efforts focused on elucidating alpha satellite structure helped to make a full understanding of the genome and some of its most critical elements a real possibility [51,52].

## 5. Linking Physical and Genetic Maps of Human Centromeres

By the late 1990s and early 2000s, several groups had pushed forward the centromere field by producing integrated physical and genetic maps of centromere regions including chromosomes X, 5, and 12 [53,54,55]. These studies used pulsed-field gel electrophoresis to estimate physical alpha satellite array sizes and either radiation hybrid or linkage analyses to estimate genetic distance across the centromere. In addition to confirming the repression of recombination across centromeres, the integrated maps that resulted allowed for the anchoring of alpha satellite regions to existing genomic maps, and sometimes identified unique pericentric sequences that had not been represented in the human genome drafts [55].

Of the sequence assemblies around the centromere that do exist, the pericentric regions are the best characterized. Within these regions, a high proportion of segmental duplications have accumulated [44,56]. Many pericentric duplications corresponding to unmapped regions of the genome were identified using monochromosomal somatic cell hybrids and PCR or FISH with known pericentric sequences and genomic BACs that recognized paralogous sequences across the genome [56]. Genome-wide analysis of the January 2001 draft assembly further revealed pericentromeric and subtelomeric enrichment for duplicated sequences, and showed that such sequences were frequently present in unmapped or misassembled segments [57]. The discordance between FISH and BLAST results in these analyses was much higher than the genome-wide rate reported in the same year [58]. Together, these studies demonstrated the importance of elucidating highly duplicated pericentric regions in order to accurately understand the Human Genome Project’s results. More recent progress was made in assembling “inaccessible” regions by using linkage disequilibrium analysis of genetically distinct (admixed) genomes to map almost 20 Mb of sequence near centromeres [59]. As the number of admixed genomes available for analysis increases, this powerful technique is expected to reduce the gaps in the current reference assembly.

**Figure 4:**
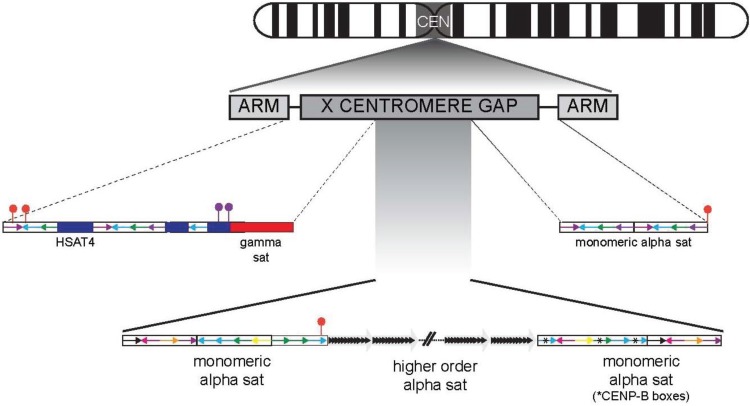
The detailed genomic organization of the human X centromere. The first contiguous genomic map of a human centromere (CEN) on the X chromosome was completed in 2001 and showed that the higher order array (large light gray arrays containing black monomer arrowheads) is flanked by unordered, monomeric alpha satellite DNA (multi-colored arrows). The regions between monomeric alpha satellite and the chromosome short (Xp) and long (Xq) arms contain other types of satellite DNA, such as gamma satellite and HSAT4. LINEs (red lollipops) and SINEs (purple lollipops) punctuate the repetitive DNA between the centromere and chromosome arms. The Xq pericentromere contains monomeric alpha satellite and a LINE element at the pericentromere-arm junction. Some of the monomers within the unordered Xq satellite contain CENP-B boxes (black asterisks). The functional significance of these monomers remains unclear.

## 6. Correlating the Genetic and Functional Centromere

In 2001, a major breakthrough in reaching beyond the boundaries of alpha satellite occurred when chromosome X short arm (Xp) genomic clones were mapped into the homogenous higher order DXZ1 array [60]. This tour-de-force used combined *in silico* and high-stringency BAC clone screening to demonstrate that even in higher order alpha satellite, enough sequence variation existed to assemble a contig extending almost half a megabase from the satellite boundary towards the centromere core that is the location of the functional centromere (Figure 4). This study revealed that heterogeneity of alpha satellite DNA increased with more distance from the DXZ1 core. These studies permitted the definition of transitions between the higher order alpha satellite and flanking regions. Monomers of alpha satellite DNA that are not ordered into multi-monomer repeat units are located directly outside of the homogenous HOR domain [9]. These monomers exhibit enough sequence variation that they can be more easily assembled and in fact represent most of the alpha satellite that exists in the human reference assembly [60,61]. The monomeric alpha satellite regions show greater sequence dissimilarity and more interspersed elements, such as L1 sequences, as they approach the chromosome arms. Currently, HSAX and HSA8 are the only human chromosomes represented in the genome assembly with contiguous sequence from higher-order alpha satellite to both arms [62,63].

Subsequent to these findings, several groups began analyzing alpha satellite at increasing sequence depth, discovering new alpha satellite polymorphisms and repeat organization. Building on the work of previous decades, targeted sequencing of several well-characterized arrays was performed. The high copy number of alpha satellite HORs on each chromosome permitted analysis of intra-homolog SNPs in addition to inter-individual variation that was paired with restriction digestion for haplotype analysis [64]. These studies revisited the molecular basis for variation within alpha satellite by pinpointing where unequal exchange occurred to produce array homogenization.

## 7. The Computational Challenge of Alpha Satellite Genome Assemblies

The bottleneck in generating alpha satellite assemblies has undoubtedly been the sophistication of assembly tools that are required to order distinguishable monomeric sequences within highly homogenous arrays. Several groups have developed *in silico* tools for analyzing higher order alpha satellite sequence available in genome assemblies [65,66]. These computational and *in silico* approaches are most effective when combined with experimental approaches that mapped clones by FISH to verify their location in or near the higher order array. Indeed, such dry/wet approaches were used to map the region spanning the Xp centromere-arm junction and to characterize the centromere of human chromosome 17 [60,61,67]. In the latter instance, a novel higher-order array (D17Z1-B) was discovered on chromosome 17 [67], emphasizing the power of this integrative approach. Another novel HOR array, localized by BLAST to HSA22 and verified by FISH to hybridize to HSA14 and 22, was found by “rescuing” unassembled alpha satellite sequence information from whole genome sequencing (WGS) repositories [68]. These studies revealed that while challenging to assemble, repetitive satellite regions, particularly in the centromere, hold a wealth of complex genomic structure and potentially functional information.

## 8. Assembling Centromeres in the Present Day

Previous studies utilized traditional sequencing technologies that have the potential to contain several 171-bp monomers per read. Next generation short-read sequencing technology has enabled the recent increase in whole-genome sequencing and the amount of human sequence information available overall. Nevertheless, short reads present a particular challenge for assembling alpha satellite sequence. It appears that this obstacle of aligning short-read alpha satellite sequences can be overcome to utilize functional information gleaned from chromatin immunoprecipitation with centromeric protein antibodies and Illumina sequencing of the DNA that is captured [51]. This ChIP-sequencing (ChIP-seq) approach utilized the reference assembly as well as the HuRef genome, first by aligning the HuRef alpha satellite reads to the reference assembly. After this alignment, the reference alpha satellite was broken into sliding windows, and the alignment checked back onto the HuRef reads to determine the “mappability” of each window. This mappability information was then used for alignment of the short Illumina reads generated by ChIP-seq. It should be noted, however, that this study did not have the means to extend beyond the edges of the reference assembly into the homogenous centromere cores (see Future Perspectives). Another major discovery from the assembly annotation of this work was that many more chromosomes than previously thought contain two or more higher order alpha satellite arrays [51,61,69,70,71]. This finding has raised the complexity of centromeres to a new level and introduced the possibility that the location of centromere assembly may be quite variable in humans. This is indeed the case for human chromosome 17 on which the centromere can be assembled at either of the two higher order repeat arrays [26]. This new information suggests that in addition to alpha satellite haplotypes, there may a number of functional centromeric genotypes. How a functional genotype might affect long-term chromosome stability is an open question.

## 9. Future Perspectives

It is now 2014, so what can we expect from the centromere field in the next decade? Based on the foundation laid by the Human Genome Project era, the most exciting areas of centromere research are in some of the following areas.

### 9.1. Centromere Assemblies

Clearly, the most significant frontier that remains to be explored in centromere biology is complete genomic centromere assemblies. With the recent advances in the past two years alone using long-template sequencing and advanced computational approaches that have sampled, annotated, and assembled centromere sequences in multiple genomes, centromere reference sequences are a real possibility. Just recently, ordering of monomer sequences from whole-genome shotgun reads has produced the first linear characterization of centromeric assemblies for alpha satellite arrays from chromosomes X and Y [72]. Increasing read lengths offered by multiple platforms offer the potential to contain several multi-kilobase HORs in one read. In fact, long PacBio reads have already accelerated the discovery and mapping of centromeric tandem repeats in a variety of species [52]. These third generation sequencing techniques should enable longer alpha satellite sequence assemblies and better understanding of centromere structure and neighboring variant HORs. Completion of even a few centromere assemblies will undoubtedly be important, but given the amount of variation in alpha satellite organization and size, the ultimate goal would be to produce centromere assemblies for each individual. These personalized maps would be useful for defining the spectrum of sequences that correlate with functional competency. In addition, they will allow identification of other features—such as genes or non-coding elements—that are present within current centromere/pericentromere gaps. These sequences may require centromeric locations for proper function, similar to heterochromatic genes in *Drosophila* [48,73]. Indeed, a human muscle disorder has been mapped to the gene KCNJ18 that is located in an assembly gap on 17p11.2 [74]. It is possible that other genes or elements within centromere regions may be associated with diseases for which the molecular basis remains undefined.

### 9.2. Centromeric Variation and Functional Capacity

The ability to confidently assemble centromeric contigs should permit identification of the full range of variability in alpha satellite, including sequence and size variants [72]. Such variation will shed light on the molecular mechanisms that regulate alpha satellite homogenization, but also effects of fundamental processes such as DNA replication and DNA repair on alpha satellite stability. Ultimately, characterization of alpha satellite variation would reveal the range of sequences that are capable of supporting centromere function. HAC studies have taught us that not all alpha satellite sequences have the capacity to support *de novo* centromere assembly [29,32]. The reasons for this have been largely unexplored, and mostly attributed to the presence or absence of CENP-B boxes in alpha satellite [33,75]. One would expect that like a given complex human disease that is often associated with various SNPs, many types of sequence variation would be associated with diminished centromere function. Complete, personalized centromeric assemblies linked to functional centromere status would expedite experiments to compare efficiencies of various sequence variants in *de novo* centromere assembly and/or centromere maintenance.

### 9.3. Maps of Functional Centromeric Domains

The consensus in the centromere field is that centromere identity is specified by epigenetic mechanisms. However, without detailed genomic information, this theory is not irrefutable. Centromere proteins, such as the histone H3-like protein CENP-A, are assembled onto alpha satellite DNA to create a specialized type of nucleosome within unique chromatin that distinguishes the centromere from the rest of the genome [76,77]. CENP-A and other proteins create a complicated network of protein sub-complexes that link the chromatin to the structural kinetochore that interacts with spindle microtubules [78]. However, chromatin that contains CENP-A nucleosomes is only assembled on a portion of alpha satellite DNA [79,80]. How and why CENP-A is recruited to only a subset of alpha satellite HOR and/or monomers is unclear. Recent studies have revealed that CENP-A nucleosomes on the human X chromosome are positioned at monomers that do not contain CENP-B boxes [81]. One could speculate that distribution of CENP-B boxes within an alpha satellite array and sequence variation that interrupts the CENP-B box motif or makes the motif non-functional (not bound by CENP-B) might impact CENP-A chromatin assembly and centromere function. Complete centromeric assemblies of many human chromosomes will be important for addressing this possibility experimentally.

## 10. Conclusions

**Figure 5:**
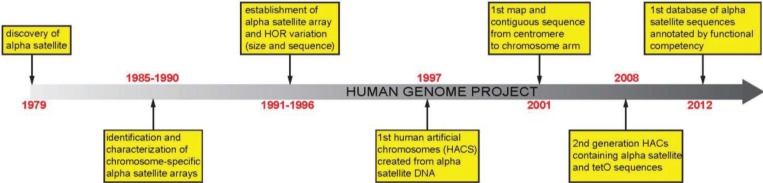
Timeline of major discoveries in human centromere genomics. Since the discovery of alpha satellite DNA in 1979, the understanding of the sequence, organization, and functional aspects of this sequence flourished during the Human Genome Project era. Recent years have shown the use of human artificial chromosomes (HACs) and the creation of the first database of alpha satellite sequences linked to their functional capacity.

Since the discovery of alpha satellite DNA in the late 1970s, the field has moved from identification of centromeric sequences at every human centromere to a basic molecular understanding of the organization and structure of alpha satellite monomers into homogeneous higher order repetitive arrays (Figure 5). The Human Genome Project was essential in providing a rough and limited reference assembly for centromeres of three chromosomes (X, 8, 17). These fundamental studies of alpha satellite DNA paved the way for pioneering functional assays in which the sequence was tested in *de novo* centromere assembly in human artificial chromosome assays. HACs have been the gold standard for testing centromere assembly, but are now being used to explore chromosome stability and gene expression. The next challenge will be to complete genomic assemblies for all human centromeres in multiple individuals and populations and to develop the next generation of functional assays to test the role of alpha satellite variation in centromere function, chromosome stability, and disease association.
